# Roles of Gibberellins and Abscisic Acid in Regulating Germination of *Suaeda salsa* Dimorphic Seeds Under Salt Stress

**DOI:** 10.3389/fpls.2015.01235

**Published:** 2016-01-13

**Authors:** Weiqiang Li, Shinjiro Yamaguchi, M. Ajmal Khan, Ping An, Xiaojing Liu, Lam-Son P. Tran

**Affiliations:** ^1^Center for Agricultural Resources Research, Institute of Genetics and Developmental Biology, Chinese Academy of ScienceShijiazhuang, China; ^2^Signaling Pathway Research Unit, RIKEN Center for Sustainable Resource ScienceTsurumi, Japan; ^3^Department of Biomolecular Sciences, Graduate School of Life Sciences, Tohoku UniversitySendai, Japan; ^4^Qatar Shell Professorial Chair for Sustainable Development, Centre for Sustainable Development, College of Arts and Sciences, Qatar UniversityDoha, Qatar; ^5^Arid Land Research Center, Tottori UniversityHamasaka, Japan; ^6^Plant Abiotic Stress Research Group & Faculty of Applied Sciences, Ton Duc Thang University, Ho Chi Minh City, Vietnam; Signaling Pathway Research Unit, RIKEN Center for Sustainable Resource ScienceTsurumi, Japan

**Keywords:** germination, *Suaeda salsa*, gibberellins, abscisic acid, dimorphic seeds, salt stress

## Abstract

Seed heteromorphism observed in many halophytes is an adaptive phenomenon toward high salinity. However, the relationship between heteromorphic seed germination and germination-related hormones under salt stress remains elusive. To gain an insight into this relationship, the roles of gibberellins (GAs) and abscisic acid (ABA) in regulating germination of *Suaeda salsa* dimorphic brown and black seeds under salinity were elucidated by studying the kinetics of the two hormones during germination of the two seed types with or without salinity treatment. Morphological analysis suggested that brown and black are in different development stage. The content of ABA was higher in dry brown than in black seeds, which gradually decreased after imbibition in water and salt solutions. Salt stress induced ABA accumulation in both germinating seed types, with higher induction effect on black than brown seeds. Black seeds showed lower germination percentage than brown seeds under both water and salt stress, which might be attributed to their higher ABA sensitivity rather than the difference in ABA content between black and brown seeds. Bioactive GA_4_ and its biosynthetic precursors showed higher levels in brown than in black seeds, whereas deactivated GAs showed higher content in black than brown seeds in dry or in germinating water or salt solutions. High salinity inhibited seed germination through decreasing the levels of GA_4_ in both seeds, and the inhibited effect of salt stress on GA_4_ level of black seeds was more profound than that of brown seeds. Taken together higher GA_4_ content, and lower ABA sensitivity contributed to the higher germination percentage of brown seeds than black seeds in water and salinity; increased ABA content and sensitivity, and decreased GA_4_ content by salinity were more profound in black than brown seeds, which contributed to lower germination of black seeds than brown seeds in salinity. The differential regulation of ABA and GA homeostases by salt stress in dimorphic seeds might provide a strategy for *S. salsa* plants to survive adverse environmental conditions.

## Introduction

*Suaeda salsa*, a leaf succulent annual herb in Chenopodiaceae family, is a main halophyte species in saline soils of China, which has economical value as being a valuable source of oil, vegetable and fodder (Zhao et al., 2002; Xu et al., 2013; Song and Wang, 2015). This species was also named as *S. maritima* subsp. *salsa* (L.) Soó or *S. liaotungensis* Kit by other reports (Li et al., 2014; Wang et al., 2015). Seed germination of halophytes is a critical stage for population establishment in saline soil and the inner conditions of seeds (Khan and Ungar, 1984; Gul et al., 2013). Like other plant species, the best germination of halophytes is obtained under non-saline conditions, and their germination decreases with the increase in level of salinity (Khan and Ungar, 1984; Gul et al., 2013). High salinity inhibits seed germination by either restricting the supply of water (osmotic effect) or causing specific injury to the metabolic machinery through disturbing the ionic balance (ionic effect) (Bajji et al., 2002; Gul et al., 2013). Thus, for the successful establishment of plants in saline environments, seeds must remain viable at high salinity and germinate when salinity decreases (Gul et al., 2013).

Abscisic acid (ABA) and gibberellins (GAs) are well-known phytohormones that are involved in regulating seed germination in *Arabidopsis*. These two hormones regulate seed germination in opposite manner. While ABA inhibits seed germination, GAs promote this biological process (Finkelstein et al., 2008; Piskurewicz et al., 2008; Yamaguchi, 2008; Nambara et al., 2010). It was reported that ABA biosynthetic mutants can germinate in the presence of salt, and fluridone (an inhibitor of ABA biosynthesis) can alleviate germination of various plants under salinity (Cheng et al., 2002; Ruggiero et al., 2004), suggesting that ABA biosynthesis might be affected by salinity during germination. With regard to GAs, bioactive GA_4_ is synthesized by two enzymes GA20ox and GA3ox (GA 20-oxidase and GA 3-oxidase) from GA_12_ in *Arabidopsis*. GA20ox converts GA_12_ to GA_15_, then to GA_24_, and finally to GA_9_ that is subsequently converted to GA_4_ by GA3ox. On the contrary, GA_9_ and GA_4_ are catalyzed to GA_51_ and GA_34_, respectively, by GA2ox (GA 2-oxidase) (Hedden and Thomas, 2012). Previous studies in *Arabidopsis* have showed that salt stress up-regulated genes involved in GA inactivation (e.g., *GA2ox7* gene) and suppression of GA signaling (e.g., DELLA proteins encoding genes) (Achard et al., 2006; Kim et al., 2008; Magome et al., 2008; Yuan et al., 2011; Colebrook et al., 2014). However, little is known about the effects of salt stress on the biosynthesis of the active GA_4_.

It has been demonstrated that seed polymorphism provides an adaptive advantage in saline habitats through the production of multiple germination periods, which increases the chances of survival of at least some seedling cohorts (Imbert, 2002; Matilla et al., 2005). The two seed morphs of *S. salsa* (brown and black seeds) can provide multiple germination cohorts in saline habitats, providing opportunities for this species to establish next generation (Khan et al., 1998; Li et al., 2008). However, the regulatory functions of these two hormones in differential germination of dimorphic seeds under salt stress remains elusive.

Thus, in this study we examined the effects of salinity on the GA and ABA metabolism during seed germination to assess the roles of these two hormones in regulating germination of dimorphic seeds of *S. salsa* under salinity. Our findings showed that dimorphic seeds showed significant differences in the content of ABA and GAs, as well as in the sensitivity to ABA. Furthermore, our results suggest that salinity differentially inhibits germination of dimorphic seeds by differentially affecting the level of endogenous ABA and GA_4_ in the seeds, as well as the sensitivity of the seeds to ABA.

## Results

### Visible Events During Dimorphic Seed Germination

**FIGURE 1:**
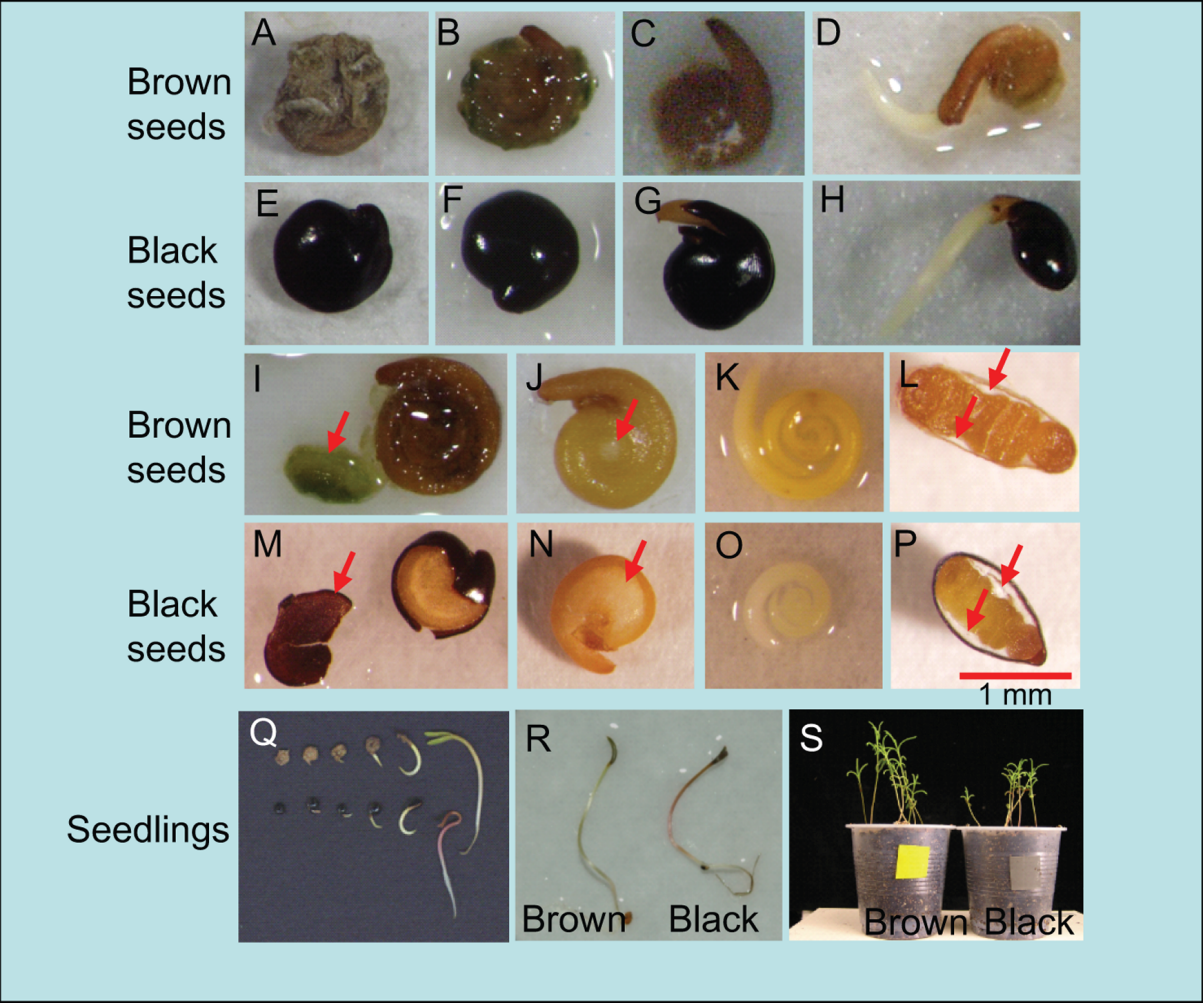
**Morphology of dimorphic seeds (black and brown) and seedlings of *Suaeda salsa*.** **(A–H)** Visible events during the incubation of *S. salsa* brown and black seeds in water; **(A,E)** Dry brown and black seeds; **(B,C,F,G)** Brown and black seeds in water for 10 min and 10 h; **(D,H)** Radicle emergence through the seed covering layers (seed coat and endosperm) signals the completion of germination after 24 h of imbibition in water. **(I,M)** Outer coat was partly removed from brown and black seeds after 10 min of imbibition in water. Red arrows indicate outer coat removed from seeds. **(J,N)** The embryo and endosperms are visible after outer coat of brown and black seeds was fully removed. Red arrows indicate endosperms. **(K,O)** Removal of inner coat after 10 min of imbibition in water showed coiled embryo of brown and black seeds. **(L,P)** Section through dry black and brown seeds. Red arrow indicate endosperms. **(Q)** Distinct stages of *S. salsa* brown (upper) and black (lower) seeds during germination, and developed seedlings after 0, 6, 12, 24, 36, and 48 h of imbibition in water. **(R)** 3-day-old seedlings from brown and black seeds. **(S)** 3-week-old seedlings from brown and black seeds.

Brown seeds are enclosed by brittle outer coat, thus absorb water quickly (**Figures 1A–D,I,L**), while black seeds are enclosed by outer layer of the thin coat, thus they show hard, waxy and un-wettable appearance (**Figures 1E–H,M,P**). Both seeds have a brown inner coat on the underside (**Figures 1J,N**), and black seeds contain more endosperms than brown seeds (**Figures 1J,L,N,P**). The embryo of brown seed is bigger than black seeds with brown in color, while that of black seed is white (**Figures 1K,O**).

For black seeds, black outer coat opening (**Figure 1G**) was the first visible event, leading to radicle protrusion (germination, **Figure 1H**) of inner coat when absorbing water. For brown seeds, the process was not so clear because the outer coat was very soft after imbibition (**Figures 1C,D**). Seedlings from dimorphic seeds are similar in morphological features (**Figure 1Q**). Three-day-old seedlings germinated from black seeds were slightly smaller than those from brown seeds (**Figure 1R**). Hypocotyl and cotyledons of seedlings from black seeds were slightly redder than those from brown ones (**Figures 1Q,R**). After growing for 3 weeks in the growth chamber, there was significant difference in height and appearance between the seedlings germinated from two types of seeds (**Figure 1S**). Seedlings from brown seeds were longer (3.76 ± 0.16 cm) than those from black seeds (3.18 ± 0.22 cm).

### Germination of Dimorphic Seeds in Response to ABA Under Water and Salinity

**FIGURE 2:**
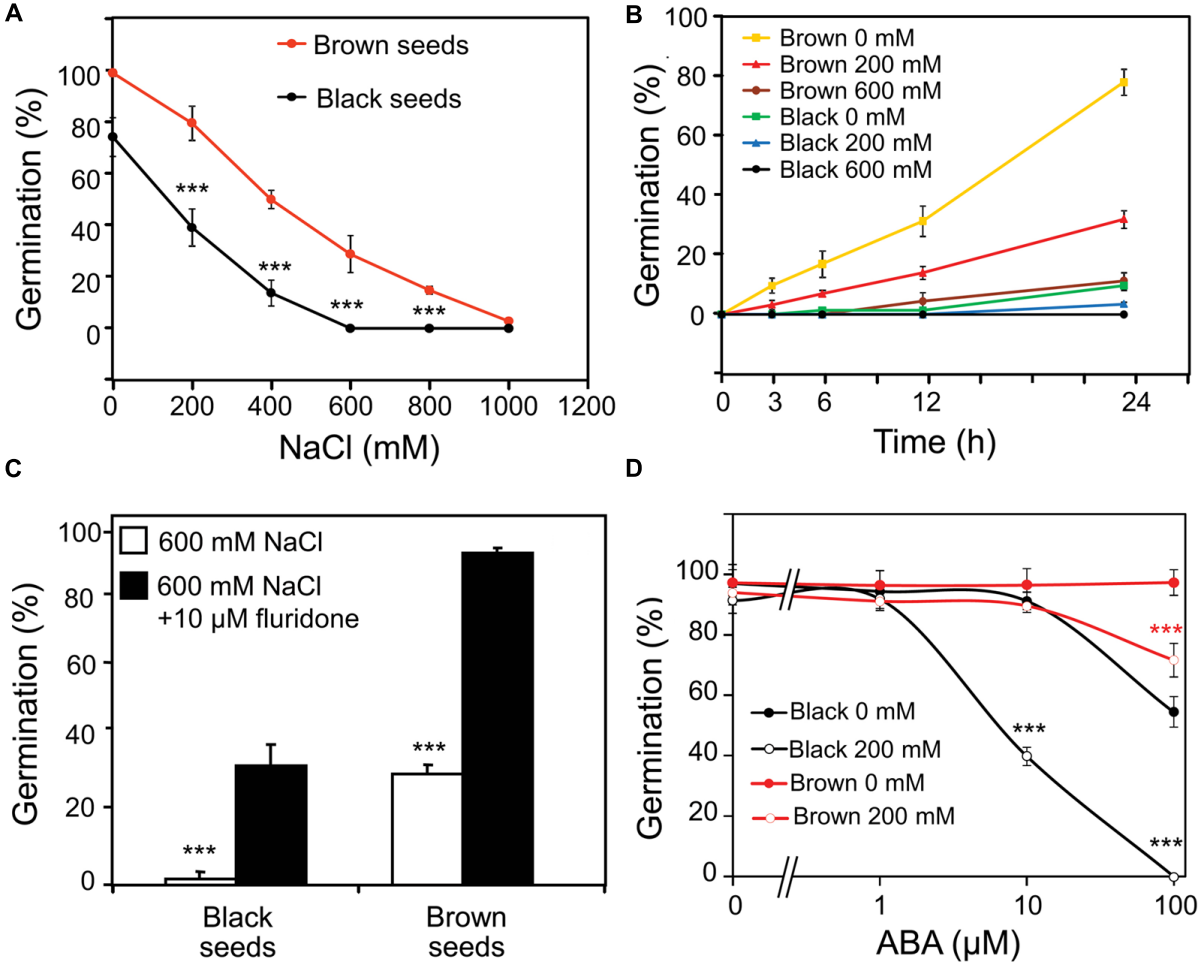
**Effect of ABA on germination of *Suaeda salsa* brown and black seeds under normal and salt stress conditions.** **(A)** Germination percentage of brown and black seeds of *S. salsa* treated with 0, 200, 400, 600, 800, or 1000 mM NaCl solution. **(B)** Germination percentage of brown and black seeds of *S. salsa* at 3, 6, 12, and 24 h after imbibition in 0, 200, or 600 mM NaCl solution. **(C)** Germination percentage of brown and black seeds of *S. salsa* treated with 600 mM NaCl solution in the presence or absence of 10 μM fluridone. **(D)** Germination percentage of brown and black seeds of *S. salsa* treated with 0 or 200 mM NaCl solution in the presence of 10 μM fluridone and 1, 10, and 100 μM ABA. The brown and black seeds were treated with 0, 200, 400, 600, 800, or 1000 mM NaCl solution under continuous light at 22°C, and germination percentages were determined after 5 days of treatment. Percentage means and SE values (error bars) were calculated from the results of three independent experiments (*n* > 100 seeds/seed type/experiment). Asterisks indicate significant differences as determined by a Student’s *t*-test (^∗∗∗^*P* < 0.001).

Brown seeds showed higher germination percentage than black seeds in water or NaCl solution (**Figure 2A**). Germination of brown seeds was earlier and faster than that of black seeds in both water and NaCl solutions (**Figure 2B**). With the increase in concentration of NaCl, the onset of germination delayed and the germination percentage decreased for both types of seeds (**Figure 2B**). In the presence of 600 mM NaCl, germination percentage of black seeds was almost 0%, whereas approximately 30% of brown seeds were able to germinate (**Figures 2A,B**).

To examine whether the synthesis of endogenous ABA in germinating seeds was affected by salinity which in turn influenced the germination percentage, fluridone (a potent inhibitor of ABA biosynthesis) was first used in a germination assay under salt stress. As shown in **Figure 2C**, addition of 10 μM fluridone rescued the germination of brown and black seeds of *S. salsa* in the presence of 600 mM NaCl, suggesting that ABA biosynthesis might be induced by salinity in germinating seeds, which would lead to inhibition of germination. To verify this hypothesis, both fluridone and exogenous ABA were used in a germination assay of *S. salsa* seeds with and without salt stress. As shown in **Figure 2D**, treatment of seeds with 200 mM NaCl in the presence of 10 μM fluridone and various concentrations of ABAs inhibited germination of black seeds more significantly than that of brown seeds. Additionally germination percentage of black seeds in the presence of 10 μM fluridone and various concentrations of ABA without NaCl was remarkably lower than that of brown seeds. These results suggested that black seeds are more sensitive to both exogenous ABA and salt stress-induced endogenous ABA than brown seeds.

### ABA Contents in Dimorphic Seed During Germination Under Water and Salinity

**FIGURE 3:**
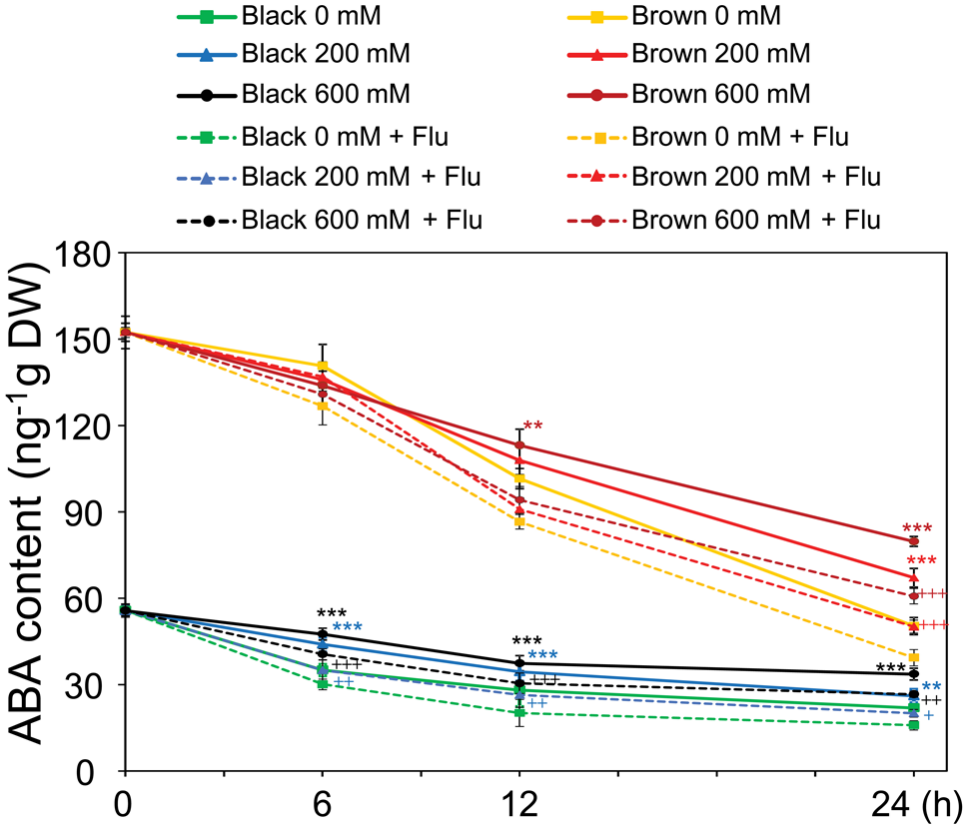
**Abscisic acid (ABA) contents in *Suaeda salsa* brown and black seeds of during germination process under normal and salt stress conditions.** The brown and black seeds were treated with 0, 200, or 600 mM NaCl solution in the presence or absence of 10 μM fluridone (Flu) under continuous light at 22°C. After 0, 6, 12, and 24 h of treatment, the seed germination was examined and seed samples were harvested to determine ABA content. Means and SE values (error bars) were calculated from the results of three independent experiments. Asterisks indicate significant differences in ABA content between salt-treated and water-treated samples in the presence of Flu (^∗∗^*P* < 0.01 and ^∗∗∗^*P* < 0.001) or in the absence of Flu (^+^*P* < 0.05; ^++^*P* < 0.01, and ^+++^*P* < 0.001) as determined by a Student’s *t*-test. DW, dry weight.

As a means to check whether the endogenous ABA content was indeed increased by salinity in germinating seeds which subsequently decreased the germination percentage, in the next line of our study ABA contents were determined in germinating dimorphic seeds under both water and salinity conditions. ABA levels were found to be higher in brown seeds than in black seeds during the time course of imbibition to water and salt solutions (**Figure 3**). Although the ABA contents gradually decreased in both types of germinating seeds, salinity treatments enhanced ABA levels during germination. Moreover, the induction effect of salt stress on ABA accumulation was much earlier in black seeds than brown seeds. For instance, ABA levels in black seeds treated with 200 and 600 mM NaCl were higher than that of water control after 6 h of imbibition, while ABA levels in brown seeds were induced by salt stress only after 12 h of treatment (**Figure 3**). Addition of fluridone to the germinating solutions further reduced the ABA contents in both brown and black seeds with or without salt stress, with higher ABA contents being observed in both seed types treated with salt stress + fluridone solution than with water control + fluridone solution (**Figure 3**). These results together might suggest that salinity inhibits germination of dimorphic seeds at least in part by inducing ABA accumulation, which happens earlier in black seeds than brown ones.

### Germination of Dimorphic Seeds in Response to GA_1_ and GA_4_ Under Water and Saline Conditions

**FIGURE 4:**
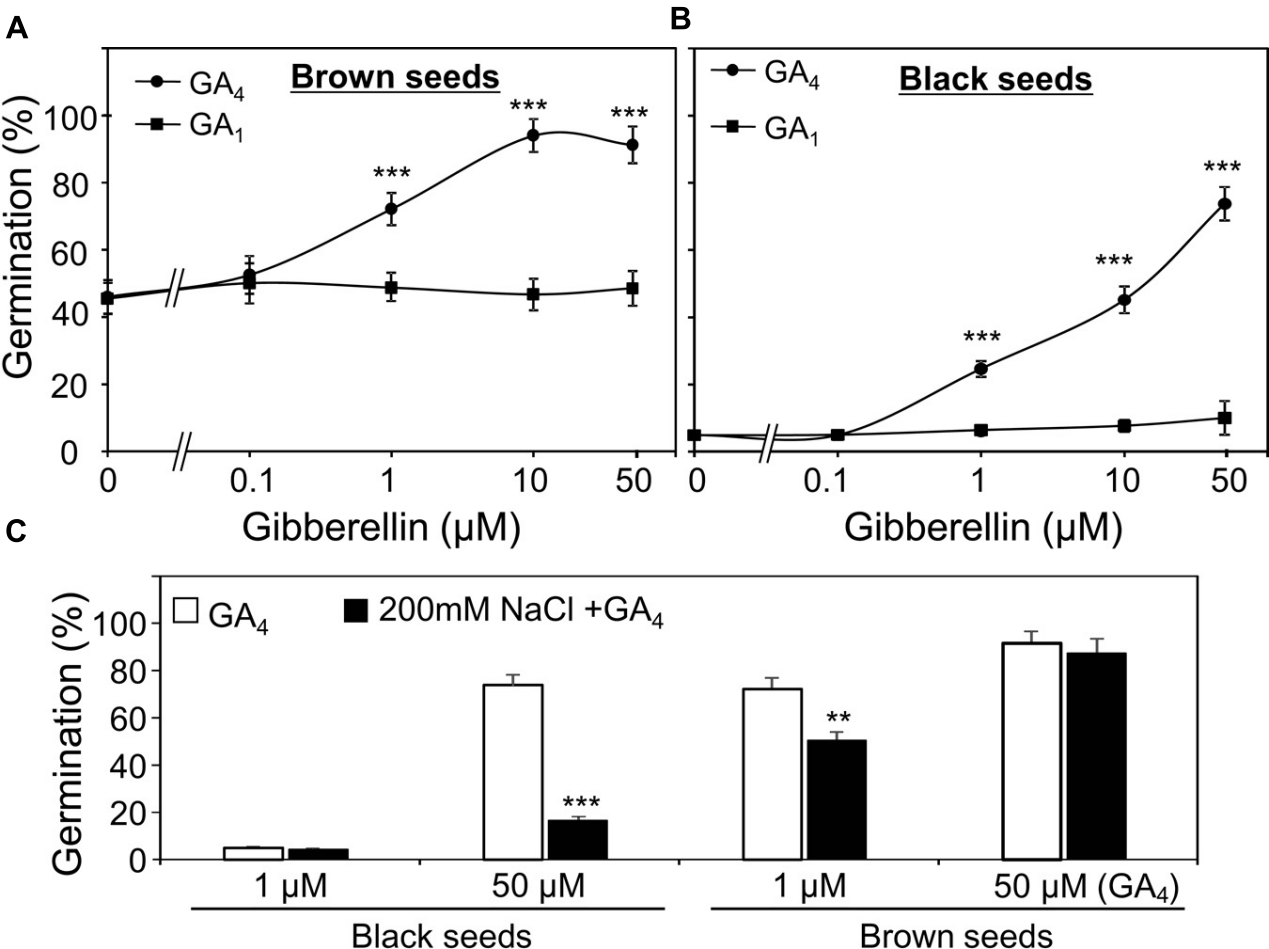
**Effect of gibberelline (GA) on germination of *Suaeda salsa* brown and black seeds under normal and salt stress conditions.** Germination percentage of brown **(A)** and black **(B)** seeds in the present of 50 μm paclobutrazol and indicated concentrations of GA_4_ or GA_1_ under non-stressed conditions. The brown and black seeds were treated with 0, 0.1, 1, 10, or 50 μM GA_1_ or GA_4_ solution containing 50 μM paclobutrazol under continuous light at 22°C. Germination percentages were determined after 5 days of treatment. **(C)** Germination percentage of brown and black seeds in the present of 50 μm paclobutrazol and indicated concentrations of GA_4_ under salt stress conditions. The brown and black seeds were treated with 1 or 50 μM GA_4_ solution containing 50 μM paclobutrazol with or without 200 mM NaCl under continuous light at 22°C. Germination percentages were determined after 5 days of treatment. Percentage means and SE values (error bars) were calculated from the results of three independent experiments (*n* > 100 seeds/seed type/experiment). Asterisks indicate significant differences as determined by a Student’s *t*-test (^∗∗^*P* < 0.01 and ^∗∗∗^*P* < 0.001).

GA_1_ and GA_4_ are two active forms of GAs in many plants (Yamaguchi, 2008). First, to examine which form is more important for the germination of *S. salsa* seeds under water and high salinity conditions, we evaluated the promotion effects of GA_1_ and GA_4_ on the germination of dimorphic seeds in the presence of GA biosynthesis inhibitor paclobutrazol (50 μM). As shown in **Figures 4A,B**, 1 μM GA_4_ significantly promoted brown and black seed germination, while GA_1_ did not even at concentration of 50 μM. This result demonstrated that GA_4_ was more active than GA_1_ in promoting germination of both brown and black seeds treated with water, suggesting that both seed types are more sensitive to GA_4_ than GA_1_.

Thus, in the next line of our study, we investigated the promotion effect of GA_4_ on the germination of the two seed types under salt stress. In the presence of NaCl and paclobutrazol, results indicated that salt stress reduced the promotion effect of GA_4_ on germination percentage of both two seed types, which was found to be more severe on black seeds than brown seeds (**Figure 4C**). These results suggested that salinity decreased GA_4_ response of both black and brown seeds and the negative effect of salt stress on GA_4_ response of black seeds was more profound than on that of brown seeds.

### GA Contents in Dimorphic Seeds During Germination Under Water and Salinity

**FIGURE 5:**
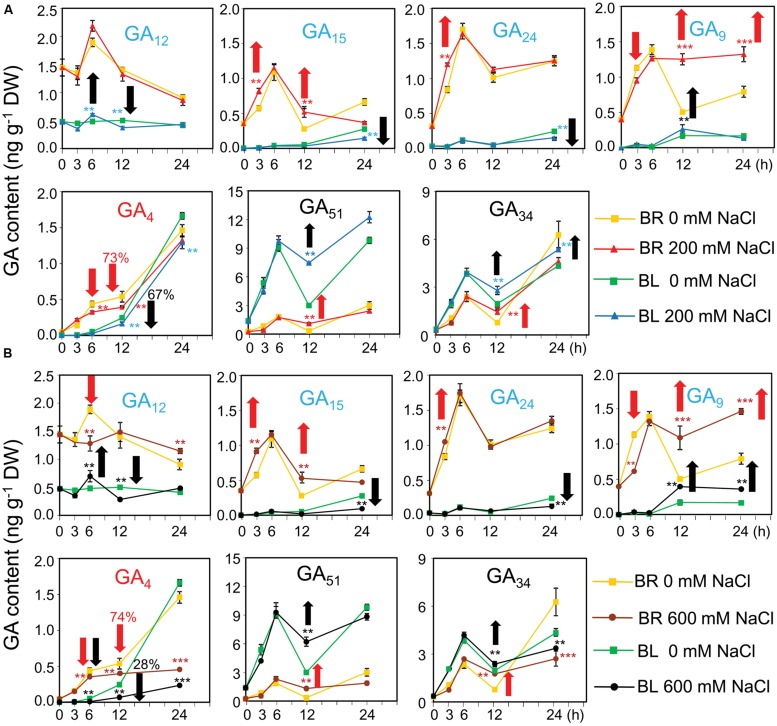
**Gibberelline (GA) contents of brown and black seeds of *Suaeda salsa* during germination process under normal and salt stress conditions.** The brown (BR) and black (BL) seeds were treated with **(A)** 0 and 200 or **(B)** 0 and 600 mM NaCl solution under continuous light at 22°C. After 0, 3, 6, 12, and 24 h of treatment, seed samples were harvested to determine the GA contents. GA_12_, GA_15_, GA_24_, and GA_9_ (blue color) are biosynthetic precursors of bioactive GAs. GA_4_ (red color) is a bioactive GA form, whereas GA_51_ and GA_34_ (black color) are deactivated forms. Red and black arrows indicate responses of brown and black seeds to salinity, respectively. Upward and downward arrows indicate increase and decrease of GA contents by salt stress, respectively. Numbers above the arrows indicate the GA reduction percentage by salt stress. Means and SE values (error bars) were calculated from the results of three independent experiments. Asterisks indicate significant differences as determined by a Student’s *t*-test (^∗∗^*P* < 0.01 and ^∗∗∗^*P* < 0.001). DW, dry weight.

To check whether the GA content were affected by salinity which subsequently altered the germination percentage, GA contents were determined in germinating dimorphic seeds under both water and salinity conditions. In dry seeds, the contents of the bioactive GA_4_ and its biosynthetic precursors (GA_12_, GA_15_, GA_24_, and GA_9_) were higher in brown seeds than black seeds (**Figure 5A**; 0 h). On the contrary, the levels of deactivated GAs (such as GA_51_ and GA_34_) were higher in black seeds than brown seeds (**Figure 5A**; 0 h).

After imbibition in water, the levels of GA_4_ biosynthetic precursors (GA_12_, GA_15_, GA_24_, and GA_9_) in brown seeds quickly increased and reached the peak at 6 h, followed by a decrease. Addition of 600 mM NaCl into germination solution significantly inhibited GA_9_ and GA_12_ syntheses in brown seeds after 3 and 6 h of treatment, respectively (**Figure 5B**). However, salt stress increased the levels of GA_15_ and GA_24_ after 3 h of treatment, as well as induced the accumulation of GA_9_ after 12 h of exposure to both 200 and 600 mM NaCl (**Figures 5A,B**). On the other hand, the levels of GA_4_ maintained the increasing tendency in brown seeds treated with water during the time course of treatment, whereas excessive salt of either 200 or 600 mM reduced the GA_4_ accumulation (**Figures 5A,B**). Similar to what was observed in brown seeds, the levels of GA_4_ biosynthetic precursors in black seeds increased in water after imbibition (**Figure 5A**). However, this increase showed slower tendency in black seeds than brown seeds. For example, content of GA_9_ in black seeds reached the peak at 12 h, while that in brown seeds was maximum at 6 h after being treated in water (**Figure 5A**). Addition of NaCl into germination solution significantly inhibited GA_12_ and GA_15_ and GA_24_ syntheses in black seeds after 12 h, and 24 h of treatment, respectively (**Figures 5A,B**). However, salt stress induced the accumulation of GA_12_ after 6 h and GA_9_ after 12 and 24 h of exposure to NaCl (**Figures 5A,B**). On the other hand, the levels of GA_4_ maintained the increasing tendency in black seeds treated with water during the time course of treatment, whereas salt stress reduced the GA_4_ accumulation. More specifically, salt stress more significantly reduced GA_4_ levels in black than brown seeds. For instance, 600 mM NaCl reduced GA_4_ content of brown seeds to 74% comparing with water control, while to 28% in black seeds at 12 h after imbibition (**Figure 5B**). In addition, germinating black seeds always contained lower levels of biosynthetic precursors and GA_4_ than brown seeds during germination within the period of 24 h after imbibition (**Figures 5A,B**). These data collectively suggested that (i) brown seeds were more active than black seed in biosynthesis of GA_4_ before and after imbibition in water, (ii) salt stress inhibited the biosynthesis of GA_4_, consequently resulting in accumulation of its precursors in both black and brown seeds, and (iii) salt stress more significantly reduced the levels of GA_4_ in black than brown seeds comparing with water control.

Although black seeds contained lower levels of biosynthetic precursors and GA_4_ than brown seeds, the levels of deactivated GAs (GA_51_ and GA_34_) were higher in black seeds than brown seeds after imbibition in water. High salinity significantly increased the levels of GA_51_ and GA_34_ than water in germinating black and brown seeds after 12 h of imbibition. These results suggested that black seeds were more active than brown seeds in deactivating GAs, and NaCl stress promoted deactivation of GAs in both black and brown seeds.

## Discussion

More than 200 species have been identified to have seed polymorphism/dimorphism with differences in seed coat properties, seed size, germination percentage and/or dormancy (Imbert, 2002; Baskin et al., 2014). Our results showed than the *S. salsa* dimorphic seeds differ not only in seed coat properties (**Figures 1I,M**) and germination ability, but also in embryo color (**Figures 1K,O**), endosperm thickness (**Figures 1J,L,N,P**) and their seedling growth (**Figures 1Q–S**). Furthermore, our study indicated the existence of the endosperms in both black and brown seeds, which was not reported by recent reports (Song et al., 2008; Song and Wang, 2015). The hard, waxy and un-wettable outer coat (**Figures 1F,M**) may prevent entry of water into embryo (Li et al., 2005; Wang et al., 2012), resulting in delay in embryo growth and thus germination (**Figures 2A,B**). Black seeds contain more endosperms than brown seeds, which might also inhibit embryo to absorb water, and thus preventing or delaying germination. In addition to the differences in seed coat morphology, embryos and seedlings form black seeds displayed different in size and color phenotype than those from brown seeds (**Figures 1K,O,Q,R**), suggesting that the embryos and seedlings form black seeds might contain different levels of pigments as compared with those from brown seeds, which would be interesting to further investigate in future. Taken together, the observation that brown seeds had larger seed size, especially embryo size and color, and better developed embryo (especially cotyledon, **Figures 1K,O**) than black seeds implied that the black and brown seeds were in different development stage, when they were formed in their mother plants, which might result in difference in their germination ability, seedling growth, and perhaps stress tolerability as well.

In the present study, we showed that the inhibition of germination of both black and brown seeds by salt stress (**Figures 2A,B**) coincided with the enhanced ABA biosynthesis (**Figure 3**), which was evidenced by a germination assay under salt stress in the presence of fluridone with and without ABA (**Figures 2C,D**). The data indicated that fluridone alleviated the negative effect of salinity on *S. salsa* seed germination (**Figure 2C**), suggesting that salt stress inhibits germination by inducing ABA synthesis, as reported in a number of plant species, such as *Arabidopsis* and rice (Cheng et al., 2002; Xiong and Zhu, 2003; Gianinetti and Vernieri, 2007). In addition, brown seeds showed higher germination percentage and faster germination than black seeds, which might be attributed to their insensitivity to ABA in comparison with black seeds. As showed in **Figure 2D**, even 100 μM ABA could not inhibit brown seed germination in water, whereas this concentration of ABA effectively inhibited germination of black seeds by approximately 50%. Salinity increased ABA response in both black and brown seeds, with higher ABA sensitivity observed in black seeds relative to brown seeds in the present of salinity (**Figure 2D**), which might be related to differential function of protein(s) homologous to ABI5 (ABA insensitive 5) of *Arabidopsis* (Yuan et al., 2011), resulting in lower germination percentage of black seeds than brown seeds under salinity (**Figure 2D**). Our results collectively demonstrate that (i) ABA sensitivities of black and brown seeds are different, and (ii) salinity increases the differential ABA responses of these two seed types, perhaps by differentially inducing endogenous ABA level, thereby differentially affecting germination of dimorphic seeds under salinity.

Although brown seeds germinated faster and showed higher germination percentage than black seeds in water and salinity (**Figures 2A,B**), surprisingly, brown seeds were found to contain higher level of ABA than black seeds (**Figure 3**). This finding was also supported by a previous study, in which the authors used the enzyme-linked immunosorbent assay to determine the ABA content (Wang et al., 2015). One possible explanation for the faster and higher germination percentage of brown seeds relative to black seeds is that brown seeds were insensitive to ABA (**Figure 2D**). Furthermore, the higher ABA content observed in brown seed type might contribute to make the brown seeds larger than black seeds (Li et al., 2008; Wang et al., 2015), because ABA promotes synthesis of seed storage proteins and lipids (Finkelstein et al., 2002; Kim et al., 2014). This finding also strengthens that brown and black seeds are in different developmental stages on *S. salsa* plants (**Figure 1**). Additionally, measurement of endogenous ABA content indicated that salt stress more significantly increased ABA content in black than brown seeds at early germination stage (**Figure 3**), which might lead to delay or inhibition of germination of black seeds in comparison with brown seeds (**Figures 2A,B**). Our finding suggests that the induction rate of endogenous ABA content in *S. salsa* dimorphic seeds by salt stress has more effects than the absolute ABA content on dimorphic seed germination during salt stress.

**FIGURE 6:**
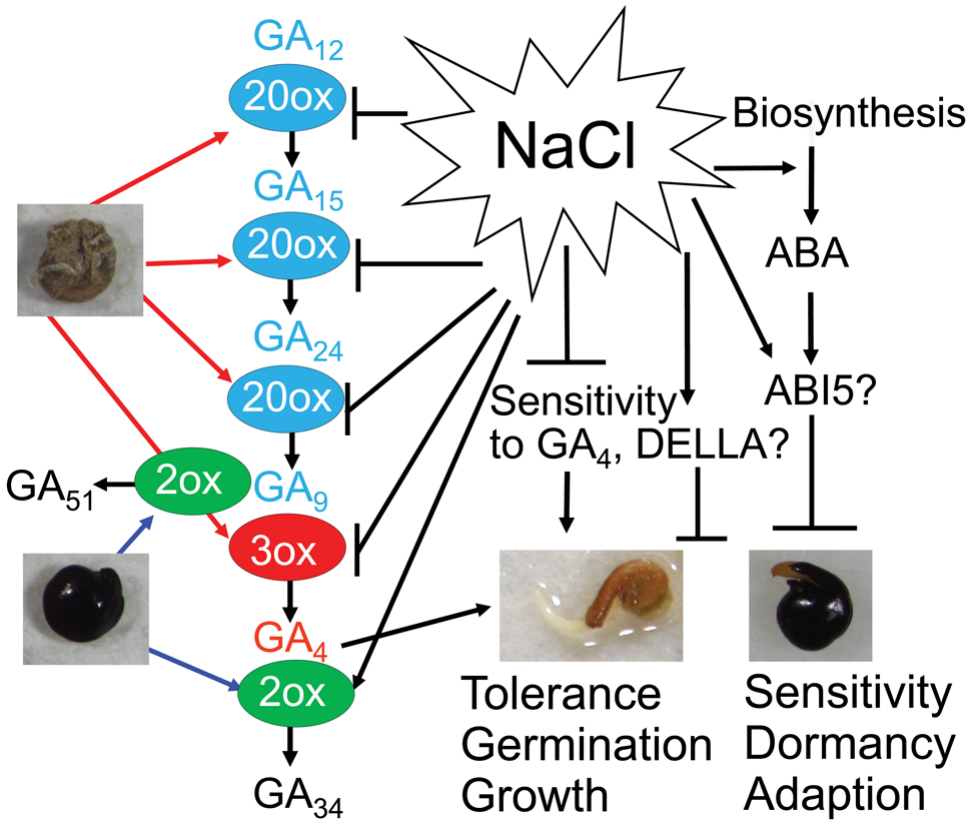
**Possible mechanisms regulating *Suaeda salsa* dimorphic seed germination under salt stress.** GA_4_ (red color) is synthesized from precursors GA_12_, GA_15_, GA_24_ and GA_9_ (blue color) by GA20ox (20ox) and GA3ox (3ox) enzymes. Bioactive GA_4_ is deactivated to inactive form GA_51_ and GA_34_ (black color) by GA2ox (2ox) enzyme. Brown seeds are more active in GA biosynthesis and contain higher content of GA precursors and bioactive GA_4_ than black seeds. Black seeds are more active in deactivation of GA_4_ and contain higher content of inactive GAs than brown seeds. Salt stress inhibits GA biosynthesis and promotes GA deactivation in both brown and black seeds. In addition, salt stress promotes ABA biosynthesis and increase ABA response. Salinity decreases sensitivity to GA_4_ probably through affecting the function of DELLA proteins. Brown seeds have better salt tolerance, and faster germination and growth rates than black seeds. On the other hand, dormancy of black seeds might be an adaptation strategy that enables the seeds to avoid adverse environmental conditions. Bars indicate negative regulation, while arrows indicate positive regulation.

In contrast to ABA, GAs have been known to play positive regulatory role in seed germination (Finkelstein et al., 2008; Nambara et al., 2010; Shu et al., 2015). In the present study, by using the GA biosynthesis inhibitor paclobutrazol in the germination assays, GA_4_ was shown to be the more biological active form than GA_1_ in the *S. salsa* seed germination (**Figure 4**), which was supported by previous studies in which germination assays were performed without paclobutrazol (Li et al., 2005, 2008). We observed that salinity decreased GA_4_ sensitivity of both black and brown seeds (**Figure 4C**) and the negative effect was more profound in black than brown seeds (**Figure 4C**), which might contribute to differential germination of dimorphic seeds under salinity. These results imply that GA response-related proteins might differentially regulate GA responses in black and brown seeds during germination under salt stress. As showed in **Figure 6**, the downstream factors of GA signaling pathway, such as *S. salsa* homologs of *Arabidopsis* DELLA proteins, might be candidate regulators as the *Arabidopsis* quadruple *della* mutant was shown to be sensitive to salt stress during germination (Achard et al., 2006; Colebrook et al., 2014). It would be interesting to carry out further genetic studies in *S. salsa* in future to gain more understanding of the roles of ABA and GA signaling pathways in regulation of the germination of black and brown seeds. Sequencing of *S. salsa* genome and development of its mutant resource should be taken to advance genetic studies in this important halophyte plant.

Analysis of the metabolism of GA_4_ in both black and brown seeds during germination strengthens the positive role of GA_4_ in promoting dimorphic seed germination in water and salt solutions. Dry and germinating brown seeds contained higher levels of GA_4_ than black seeds (**Figure 5**), which was consistent with the finding that brown seeds displayed quicker and higher germination percentage than black seeds under water, excessive salt and excessive ABA conditions (**Figures 2A–D**). In addition, higher levels of GA_4_ precursors in germinating brown than black seeds under water and salt stress treatments (**Figures 5A,B**) implied that GA biosynthesis activity is higher, perhaps due to the enhanced activities GA biosynthesis enzymes (e.g., GA20ox and GA3ox), in germinating brown than black seeds, which also supports the differential development stages of the two seed types (**Figure 1**). On the contrary, deactivated GAs forms from GA_9_ and GA_4_ (e.g., GA_51_ and GA_34_) were higher in germinating black seeds than brown seeds in water and salt solutions (**Figure 6**), suggesting that germinating black seeds possess higher GA deactivated activities than brown seeds. These data collectively demonstrate that the germinating brown seeds have higher GA biosynthesis activity but lower inactivation activity than black seeds, which may contribute to higher germination percentage and faster germination rate of brown seeds in comparison with black seeds in water and salt solutions (**Figure 5**).

As GA_4_ strongly binds to the GA receptor and is directly involved in GA signaling, maintenance of an appropriate level of endogenous GA_4_ is the most critical for seed germination (Sun, 2008). Thus, the balance in biosynthesis and deactivation of GA_4_, which is strongly affected by salinity (Hedden and Thomas, 2012), is very important in controlling the GA_4_ homeostasis in the early stage of seed germination (Yamaguchi, 2008). Salinity more highly induces GA_34_ in black than brown seeds (**Figures 5A,B**), implying that salt stress promotes deactivation of GA_4_ through upregulation of GA-deactivating GA2ox activity in *S. salsa* in a similar process as in *Arabidopsis*, especially in black seeds (Magome et al., 2008; Colebrook et al., 2014) (**Figure 6**). Our results showed that the effects of salt stress on the levels of some GA biosynthesis precursors in both brown and black seeds were significantly different at various time points (**Figures 5A,B**), suggesting that salt stress differentially affects the biosynthesis of active GA_4_ in brown and black seeds. Salt stress decreased the content of GA_9_ and increased that of their precursors GA_15_ and GA_24_ in brown seeds after 3 h of imbibition (**Figures 5A,B**), indicating that salinity inhibited the conversion of GA_15_ to GA_24_ and GA_24_ to GA_9_ with the involvement of GA20ox (Yamaguchi, 2008), suggesting that salt stress might affect activity of GA20ox in brown seeds after 3 h of imbibition (**Figure 6**). However, there is no difference in GA content in black seeds after 3 h of imbibition in salt stress, implying that GA20ox activity in black might not be affected by salt stress at this time point. GA3ox is involved in the final step of synthesis of GA_4_ from GA_9_, and in most case, the final step is the main site for active regulation by environmental factors (Yamauchi et al., 2004). The decrease and increase in GA_4_ and GA_9_ contents, respectively, in both black and brown seeds at 12 h after imbibition in salt solution suggested that salinity inhibited the conversion of GA_9_ to GA_4_, in which GA3ox is involved (**Figure 6**). Therefore, we supposed that salinity inhibited GA3ox activity in both black and brown seeds after 12 h of imbibition in salt solution. Salinity induced lower level of GA_4_ in black than brown seeds, which might contribute to reduced germination of black seed relative to brown seeds under salinity. Collectively, our results suggest that salinity inhibits GA_4_ biosynthesis perhaps through reducing GA20ox and GA3ox activities, as well as promotes inactivation of GA_4_ probably through increasing GA2ox activity in *S. salsa* dimorphic germination seeds, resulting in a decrease in endogenous level of GA_4_ (**Figure 6**). And, this effect of salt stress is more severe on black than brown seeds.

## Conclusion

Brown and black seeds are in different development stages, when harvested from *S. salsa* plants, and thus they show different GA and ABA homeostases, resulting in their differential germination. In addition, the differential regulation of ABA and GA kinetics by salt stress observed between brown and black seeds results in lower sensitivity of brown seeds to salinity during germination in comparison with black seeds, thereby providing different adapting strategies of dimorphic seeds to saline environment.

## Materials and Methods

### Plant Materials

Seeds of *S. salsa* were collected during the fall of 2011 in the coastal saline soils of Huanghua City, Hebei Province, China. The seeds were then dried for a few days, and stored in paper bags in laboratory at approximately 20°C and 30–40% relative humidity. Black and brown seeds were manually separated from the inflorescence.

### Microscopy and Image Preparation

Black and brown seeds of *S. salsa* were incubated under standard conditions, and appropriate developmental stages were selected for taking photos.

### Germination Assays

Germination was assayed at 22°C under continuous light (fluorescent lamp with approximately 100 mmol m^-2^s^-1^ light intensity) in triplicates. Seeds were washed with 0.02% Triton X solution, rinsed with distilled water, then washed with respective working solutions. Subsequently, 50–100 seeds were placed on a double layer of wet filter papers within a plastic petri dish and closed. Seeds were scored as germinated when primary root protrusion was visible. To determine the effect of salinity on germination, NaCl solutions of 0, 200, and 600 mM were used for brown and black seeds. Seed germination was counted at 3, 6, 12, 24, and 120 h.

To evaluate the effect of ABA inhibitor fluridone on germination of dimorphic seeds under salinity, 10 μM fluridone was used in germination assays of brown and black seeds in 600 mM NaCl solution. To determine the response (sensitivity) of dimorphic seeds to ABA under salt stress, ABA was used in 0, 1, 10, and 100 μM concentrations in germination assays of brown and black seeds in water or 200 mM NaCl solutions in the presence of 10 μM fluridone. To determine the response (sensitivity) to GA_1_ and GA_4_ of dimorphic seeds in salinity, GA_1_ and GA_4_ solutions of 0, 0.1, 1, 10, and 50 μM were used in germination assays of brown and black seeds in water and 200 mM NaCl solutions in the presence of 50 μM GA biosynthesis inhibitor paclobutrazol. After 5 days of cultivation, the germination seeds were counted. To determine the average percentage of germination, more than 100 seeds were used for each treatment. The experiments were independently repeated three times.

### Analyses of Endogenous Hormone Contents

For hormone analyses, seeds were germinated under the same environmental conditions as for the germination assays. For ABA analysis 50 mg of brown and black dry seeds were incubated in 0, 200, and 600 mM NaCl solutions in the present or absence of 10 μM fluridone. Samples were then collected at 0, 6, 12, and 24 h in three replicates, immediately frozen in liquid nitrogen and kept at -80°C until analysis. For GA analysis, 500 mg of brown and black dry seeds were incubated in 0, 200, and 600 mM NaCl solutions. Samples were then collected at 0, 3, 6, 12, and 24 h in three replicates, immediately frozen in liquid nitrogen and kept at -80°C until analysis. Purification and determination of ABA and GA contents were carried out by LC–MS/MS analysis as previously described (Saika et al., 2007; Li et al., 2011).

## Author Contributions

WL conceived research. WL performed the experiments and analyzed the data. SY, PA, and XL provided research materials. WL, MK, and L-ST wrote the manuscript.

## Conflict of Interest Statement

The authors declare that the research was conducted in the absence of any commercial or financial relationships that could be construed as a potential conflict of interest.
